# DEMOGRAPHIC VARIATION IN BARRIERS TO VACCINATION AMONG OLDER ADULTS: LINKS TO VACCINATION BEHAVIORS

**DOI:** 10.1093/geroni/igad104.2918

**Published:** 2023-12-21

**Authors:** Heather Fuller, Andrea Huseth-Zosel, Bryce Van Vleet, Paul Carson

**Affiliations:** North Dakota State University, Fargo, North Dakota, United States; North Dakota State University, Fargo, North Dakota, United States; North Dakota State University, Fargo, North Dakota, United States; North Dakota State University, Fargo, North Dakota, United States

## Abstract

As older adults face increased infectious disease risks, understanding barriers to vaccination is vital. The current study examines associations between vaccination barriers, demographics, and vaccination behaviors among older adults (65+) in North Dakota, a rural state with low vaccination rates. A mailed survey, including closed- and open-ended questions regarding vaccine beliefs and behaviors was conducted with 901 older adults. Potential vaccine barriers (Disliking shots, Concerns about cost, Lacking doctor recommendation, Lacking transportation, and Uncertainty how to schedule) were rated on a four-point Likert-type scale (1=Strongly Disagree; 4=Strongly Agree). Vaccine behaviors were assessed with measures of uptake for influenza, shingles, pneumonia, and SARS-CoV-2 immunizations. While endorsement of perceived barriers was relatively low, the greatest perceived barriers were cost and disliking shots. Regarding demographic variation: lower income was associated with cost concerns, lower education and rurality were associated with lacking doctor recommendation, and lower education was associated with uncertainty how to schedule. Disliking shots was significantly associated with uptake for shingles and pneumonia. Cost concerns was associated with lower shingles uptake, whereas lacking doctor recommendation was associated with lower uptake for pneumonia and SARS-CoV-2 booster. No barriers were associated with influenza or SARS-CoV-2 initial immunization uptake. While perceived vaccination barriers were low, certain populations of older adults are more at-risk of experiencing barriers, suggesting intervention opportunities. Moreover, the relevance of barrier type varied by disease, suggesting the need for disease-specific interventions. These findings suggest that practitioners should consider the diverse factors influencing barriers to older adults’ vaccination receipt.

